# Arboreal Behavior of Captive Acacia Rats *Thallomys paedulcus* (Rodentia: Muridae)

**DOI:** 10.3390/ani16111632

**Published:** 2026-05-27

**Authors:** Dionisios Youlatos, Fani Koutsougianni, Nikolaos Evangelos Karantanis, Leszek Rychlik

**Affiliations:** 1 Department Zoology, School of Biology, Aristotle University of Thessaloniki, 54124 Thessaloniki, Greece; koutsfan@gmail.com (F.K.); nikolaosevangelos.karantanis@peopleplus.co.uk (N.E.K.); 2 Department of Pharmacology, School of Medicine, University of Thessaly, 41500 Larissa, Greece; 3 PeoplePlus Group Ltd., The Quorum, Bond Street South, Bristol BS1 3AE, UK; 4 Department of Systematic Zoology, Institute of Environmental Biology, Faculty of Biology, Adam Mickiewicz University, 61-614 Poznań, Poland; leszek.rychlik@amu.edu.pl

**Keywords:** Africa, locomotion, positional behavior, postures, rodent, savanna woodland, tree-crown

## Abstract

Arboreal locomotion in small mammals requires the integration of behavioral and morphological adaptations to negotiate complex three-dimensional environments. The present study investigates the locomotor and postural behavior of captive Acacia rats (*Thallomys paedulcus*) to evaluate their arboreal capacities. A total of 21 adult individuals were observed under controlled conditions, and behavioral data were collected from video recordings using continuous bout sampling. Substrate size, inclination, and type were recorded alongside locomotor and postural modes, and substrate preferences were assessed relative to availability. Results show that Acacia rats predominantly used arboreal substrates, with a strong preference for small-diameter and horizontal supports during both locomotion and postural activities. Quadrupedalism was the dominant locomotor mode, followed by climb and clamber, while bridge and leap were used for gap crossing. Postural behavior was mainly characterized by stand, with less frequent sit, bipedal stand, and cling. Medium and large substrates, as well as oblique and vertical supports, were generally avoided, particularly during postural behavior. These findings indicate a high degree of behavioral specialization for exploiting fine terminal branches within tree canopies. The results support the arboreal propensities of Acacia rats, with adaptations primarily expressed through behavioral selectivity rather than morphological divergence. This study contributes to understanding the ecological and functional adaptations of small arboreal mammals and highlights the importance of structurally complex habitats for their survival.

## Simple Summary

Animals that live in trees must move safely across narrow, uneven, and often unstable branches. The Acacia rat (*Thallomys paedulcus*) is a small African rodent that spends most of its life in trees, but its behavior in such environments is not well understood. In this study, we observed captive Acacia rats in a controlled setting to examine how they move and position their bodies in the arboreal habitat. We found that they prefer narrow and horizontal branches for both locomotion and postures. They primarily move by walking and climbing, and they cross gaps between branches by bridging or jumping over short distances. Larger and steeper branches were used less frequently, especially when the animals were stationary. These results suggest that Acacia rats are highly adapted to living on the outer, finer parts of tree-crowns, where food sources are more abundant and predators less common. Understanding how these animals use their environment can help guide conservation efforts, especially in fragile habitats such as savanna woodlands.

## 1. Introduction

Arboreality has evolved repeatedly across mammalian lineages and represents one of the most biomechanically demanding ecological transitions in vertebrate history [1,2,3,4,5,6,7,8,9,10,11,12,13]. Among mammals, rodents constitute one of the most ecologically diverse clades and include several independent transitions to arboreality [14,15,16]. Through arboreal activity, rodents gain access to novel, diverse food sources, manage to avoid predators, and construct and exploit secure nesting sites and refuges [17,18,19,20,21,22,23,24,25,26,27,28,29]. However, the exploitation of arboreal substrates imposes substantial biomechanical challenges: arboreal rodents must safely negotiate narrow, compliant, discontinuous and variably inclined branches, as well as wide, vertical, rigid trunks, within a three-dimensional multi-layered environment [2,30]. Consequently, successful arboreality requires the integration of morphological and behavioral adaptations that enhance balance, grasping performance, substrate selectivity, and gap-crossing capacity [2,16,31,32,33,34,35,36,37].

Within African murids, the genus *Thallomys* represents one of the most distinct arboreal radiations. The Acacia rat, *Thallomys paedulcus* (Sundevall, 1846), is small arboreal and nocturnal murid with a body length between 12 and 16 cm, tail length between 13 and 21 cm, and mean body mass of approximately 68 g [18,21,38,39,40,41]. Acacia rats are primarily associated with Southern Savanna Woodland biomes of eastern and southern Africa [18,21,38,41,42,43]. The species is strongly linked to Acacia woodlands and most particularly to specific Acacia trees (*Acacia tortilis*, *A. xanthophloea*, and *A. erioloba*), building nests several meters above ground within tree cavities and thorn-protected branch networks [18,21,44,45,46,47]. Large Acacia trees function as structural habitat islands within arid savannas, offering nesting hollows and protection from predators [47,48]. The inverted conical canopy architecture of these trees creates a steeply inclined and fine terminal branch environment which provides both refuge and access to specific food resources [21,49]. Within the African savannah, Acacia rats are closely associated with Acacia woodlands, which represent critical structural components of these ecosystems [47,48]. Acacia rats may play an important ecological role as prey, parasite hosts, and potential seed dispersers, thereby contributing to broader savannah community dynamics [18,21,38,41,42,43]. However, habitat degradation and the removal of mature trees may severely disrupt arboreal pathways, reduce nesting availability, and increase vulnerability against predators [18,21] seriously impacting the survival of the species.

Morphologically, Acacia rats possess a long tail covered with elongated tactile hairs, relatively short but robust limbs, recurved claws, broad hind feet with raised pads and short toes, and a well-developed, partially opposable fifth toe, that are functionally related to arboreal habits [18,49]. However, very little is known about the arboreal behavior of the species. Earl & Nel [17] reported increased climbing ability and suggested that the length of the tail and the plantar tubercles contribute to climbing security. More recently, Karantanis et al. [50] documented the use of slow, symmetrical diagonal gaits on smaller substrates, which are progressively substituted by faster, asymmetrical half-bounding gaits on larger substrates, enabling the safe negotiation of variable arboreal substrates.

Despite this morphological and behavioral evidence, quantitative behavioral data on substrate use, and postural and locomotor strategies in Acacia rats remain limited. Similar positional evidence is essential for evaluating the degree of arboreal specialization, as substrate size selection, inclination use, and gap-crossing behavior reflect functional adaptation to canopy structure [37,51]. Comparative data from other small arboreal rodents indicate that true arboreal specialists exploit fine terminal branches, select horizontal supports for postural stability, demonstrate competent vertical claw and grasp climbing activities, exhibit diversified positional modes suited to narrow substrates, and efficiently employ controlled gap-crossing strategies [37,51,52,53]. In contrast, scansorial or semi-arboreal rodents typically display broader substrate size tolerance and greater reliance on large or oblique supports. Similar comparisons allow the evaluation of whether Acacia rats represent generalized scansorial murids or fine-branch specialists.

In this context, the present study builds on the limited body of locomotor studies on the species [17,50] by examining the locomotor and postural behavior of captive Acacia rats under controlled conditions. The novelty of this study lies in the integration of substrate size and inclination use and availability with positional behavior frequencies. This approach allows us to assess whether Acacia rats exhibit behavioral patterns consistent with arboreal specializations. Considering the ecological connection of the species with the fine-branch Acacia canopies we expect: (i) increased use and preference of small-diameter substrates during locomotor activities, (ii) considerable use of oblique and vertical substrates associated with more acrobatic positional modes, (iii) a diverse positional repertoire with substantial rates of quadrupedal, climbing and clambering activities, (iv) use of bridging and leaping primarily across short gaps, and (v) overall substrate size/inclination selectivity exceeding random substrate availability, indicating functional specialization rather than opportunistic scansorial use. By integrating this information, we aim to clarify whether Acacia rats should be considered as a highly specialized arboreal rodent adapted to structurally complex but discontinuous savanna canopies, or as a behaviorally flexible murid occupying arboreal habitats secondarily.

## 2. Materials and Methods

The present research followed the guidelines for the treatment of animals in behavioral research and teaching [54] and complied with the regulations and legislations of the Nowe Zoo and the Adam Mickiewicz University in Poznań, as well as the ethics legislation of the Aristotle University of Thessaloniki.

**Figure 1:**
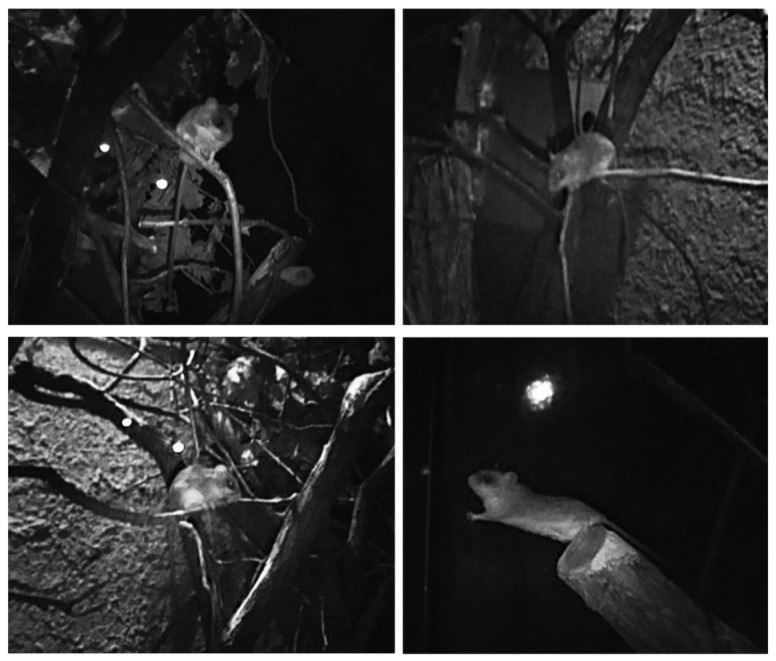
Video stills from the enclosure of *Thallomys peadulcus* showing the animals and the available substrates: sit on small oblique substrate (**top left**); stand on small horizontal substrate (**top right**); stand on medium horizontal substrate (**bottom left**); cantilever on large oblique substrate (**bottom right**).

For the purposes of the current study, we observed and filmed 12 male and 9 female captive adult Acacia rats *Thallomys paedulcus* (Sundevall, 1846) in the Nowe Zoo, Poznań, Poland. All study animals were captive-born, healthy, fully habituated to human presence, and did not display any stereotypic behaviors during the study period. Based on measurements of the male specimens (see [50]), mean head–body length was 13.4 cm (SD = 1.6 cm, range: 12.6–14.0 cm,) and mean mass was 70.2 g (SD = 11 g, range: 62–74 g). The species does not display any sexual dimorphism in body mass and dimensions [18,21]. The animals were housed together in a large enclosure (H: 190 cm x W: 300 cm x D: 140 cm) in the display colonies of the Nocturnal Pavilion of the zoo under a reversed day–night regime (Figure 1). Large glass windows made up the front and the sides of the enclosure. Additionally, the enclosure was backed by cement wall, topped by thin wire-mesh, and the floor is covered by sand. It contained a wide variety of intertwined available substrates of diverse sizes (thin twigs to wide chunks of trunks) and inclinations (horizontal to vertical), enabling the animals to move freely in a three-dimensional enriched environment. The available arboreal substrates involved mainly branches, a nest box, and several hanging feeders that assured regular and ad libitum feeding of the study animals.

Despite the enriched arboreal environment provided, substrates in an artificial enclosure are always expected to limit the locomotor and postural options of caged animals. In this case, an estimate of their availability allows for a controlled test of substrate preference or avoidance. In this way, we calculated all available substrates by unit and, subsequently, estimated the availability of the different size and inclination categories (see Table 1 for definitions). Small substrates dominated (44.3%) with medium and large ones ranking behind (29.7% and 26.0%, respectively). Regarding substrate inclination, oblique substrates were common (51.6%), followed by horizontal (28.1%) and vertical ones (20.3%). Preference or avoidance of these categories was then estimated by Jacobs’ selectivity index: D = U − A/U+ A − 2U × A, where U is the proportion of use and A is proportion of availability. Values of the index range from -1, depicting strong avoidance, to +1, showing strong preference, whereas values around 0 are considered as neutral.

The present data derive from the analysis of extensive video recordings of the study animals. For the experimental procedure, the Nowe Zoo administration granted a research permit to film the animals in situ between February and May 2013, during the days when the zoo was closed to the public. This allowed easy and uninterrupted access to the animal enclosure without external disturbance. For these administrative reasons, the animals were filmed twice per week from 10:00 to 17:00, using additional infrared lighting. Due to the reversed day–night regime of the Nocturnal Pavilion of the zoo, this period corresponded to nighttime and the animals were active. During video recording sessions we used a SONY Hi-8 CCD-TR705E camcorder (Sony Group Corporation, Minato, Tokyo, Japan) at 24 fps and at a shutter speed of 1/500th. The original Hi-8 tapes, totaling 25 h of recordings, were digitized and were subsequently analyzed frame-by-frame on a PC for data collection.

During video analysis, we used the continuous bout method for recording behavioral observations from different individual animals [55]. A bout ended when one of the recorded variables changed. Although the bout method does not guarantee the independence of behavioral events, it is most suitable for fast and agile mammals, as it can capture their behavioral diversity through the recording of rare or occasional events. During each bout we recorded: (i) substrate type, (ii) substrate size, (iii) substrate inclination, and (iv) locomotor or postural mode (Table 1). Substrate size categories were defined by the capacity of full or partial foot grasp of the animals. Finally, although positional modes and substrate use usually relate to behavioral contexts (e.g., feed, travel, etc.) in the wild, a captive setting with its spatial constraints and specific feeding conditions (i.e., hanging feeders) usually modifies and biases similar associations. Considering these limitations, we excluded behavioral contexts in the current study, despite their ecological importance in naturalistic conditions.

At the end of the sampling process, the total of collected bouts of locomotor and postural behavior was derived from the observational recording of the 21 different individual Acacia rats. As we could not identify the focal animals and there is no sexual dimorphism in the species, all the collected data for all study subjects were combined for subsequent analysis. A common problem of positional behavior studies is the autocorrelation of successive sampling events. This occurs because subsequent samples of the same individuals usually lack independence from previous observations [56]. To address this shortcoming and safely guarantee independence, we followed a trimming procedure. Initially, the complete dataset was divided into locomotor and postural subsets. Then, in each subset, we only considered every second bout (b, b + 2), deleting each intermediate one (b + 1) (see [37,57]). Following this trimming procedure, we obtained a total of 1389 bouts of locomotion and 3887 bouts of postures. Subsequently, we calculated the frequencies of the different behavioral and substrate categories for each subsample (locomotor, postural). Differences among frequencies of behaviors or substrate use were calculated using the log-likelihood G test. This test is used to determine whether observed frequencies differ significantly from expected frequencies. It is conceptually similar to a χ^2^ test, comparing observed and expected values using likelihood ratios derived from logarithms. The test is commonly applied to categorical or count data, particularly when assessing whether behaviors, substrate use, or positional frequencies occur non-randomly across categories; *p*-values ≤ 0.05 were regarded as statistically significant and only those are reported in the results section. All statistical analyses were performed in SPSS 25.0 (IBM SPSS Inc., Armonk, NY, USA).

## 3. Results

### 3.1. Substrate Type, Size, and Inclination

During behavioral observations, the captive Acacia rats primarily used arboreal substrates (94.6%) and were seldom found on the enclosure floor (5.4%). Branches were the most used arboreal substrates (82.5%), with artificial ones (12.1%) ranking behind.

Regarding substrate size, Acacia rats used extensively (Table 2) and preferred small substrates during both locomotor and postural behavior (Jacob’s selectivity index D = 0.43 and D = 0.46, respectively). Medium-sized substrates were moderately used and were used significantly more during postures than locomotion (Table 2; G = 80.2, *p* < 0.001). During both locomotor and postural behavior, medium-sized substrates were avoided (D = −0.46 and D = −0.12, respectively). Finally, large substrates were used moderately during locomotion but infrequently for postures (Table 2; G = 164.8, *p* < 0.001). They were slightly avoided during locomotion (D = −0.17) but strongly avoided (D = −0.65) during postural behavior.

In terms of substrate inclination, Acacia rats very frequently used horizontal substates (Table 2). Horizontal substrate use was significantly higher during postural than during locomotor behavior (Table 2; G = 259.8, *p* < 0.001). In both contexts, horizontal substrates were strongly preferred (locomotion D = 0.39, postures D = 0.73). On the other hand, oblique substrates were moderately used during both locomotor and postural behaviors (Table 2) and strongly avoided (locomotion D = −0.43, postures D = −0.57). Vertical substrates were moderately used during locomotion but were infrequently used for postures (Table 2; G = 289.0, *p* < 0.001). Thus, they were used according to availability during locomotion (D = 0.07) but strongly avoided (D = −0.62) during postural behavior.

### 3.2. Locomotion and Postures

**Figure 2:**
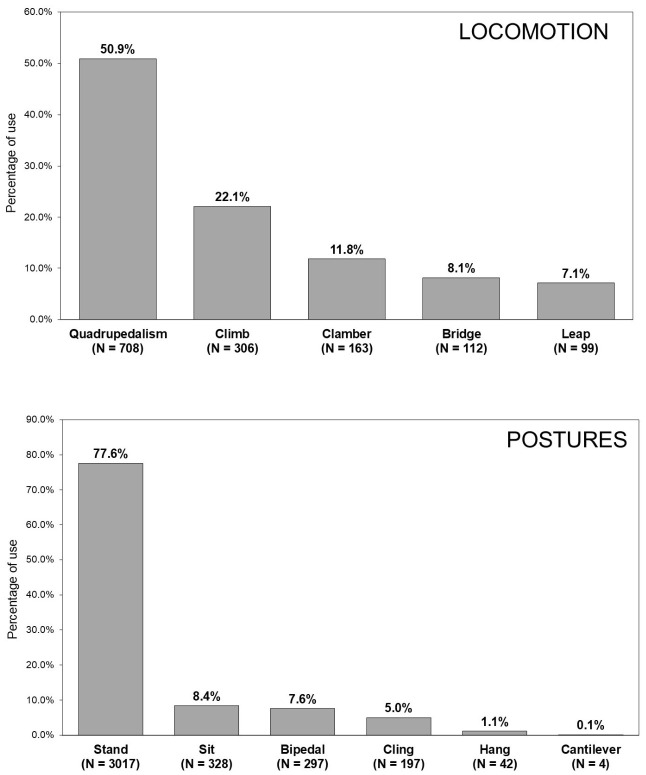
Percentages of use of locomotor (N = 1389) and postural (N = 3887) modes in captive *Thallomys paedulcus* (N below each mode corresponds to the number of bouts observed).

Quadrupedalism was the most frequent locomotor mode (Figure 2). Most quadrupedal bouts occurred on small and on horizontal substrates (Table 3). Climb was the second most frequent locomotor mode. Claw use accounted for 37.5% of climb bouts. During vertical climb, the use of small and vertical substrates dominated (Table 3; substrate size, climb vs. quadrupedalism: G = 28.7, *p* < 0.001; climb vs. clamber: G = 25.5, *p* < 0.001; substrate inclination, climb vs. quadrupedalism: G = 98.5, *p* < 0.001; climb vs. clamber: G = 28.4, *p* < 0.001). Clamber across multiple substrates was moderately used and primarily occurred on small and oblique substrates (Table 3; substrate size, clamber vs. quadrupedalism: G = 84.7, *p* < 0.001; clamber vs. bridge: G = 68.6, *p* < 0.001; substrate inclination, clamber vs. quadrupedalism: G = 94.4, *p* < 0.001; clamber vs. bridge: G = 58.4, *p* < 0.001). Of the major gap-crossing modes, bridge and leap were almost equally used (Figure 2). In either mode, small and horizontal substrates dominated, with similar rates regarding substrate size, but vertical substrates were used more during leap than bridge (Table 3; substrate inclination: bridge vs. leap: G = 4.3, *p* = 0.016).

Stand was the most frequent posture (Figure 2) and occurred mainly on small and horizontal substrates (Table 4). Sit was the second most frequent posture (Figure 2) and occurred principally on small and horizontal substrates, but at higher rates than stand (Table 4; stand vs. sit, substrate size: G = 14.7, *p* < 0.001; substrate inclination: G = 44.8, *p* < 0.001). Bipedal stand was also considerably used (Figure 2). During bipedal postures, small and horizontal substrates were frequently used (Table 4; substrate size, bipedal vs. sit: G = 30.6, *p* < 0.001; bipedal vs. stand: G = 70.7, *p* < 0.001; substrate inclination, bipedal vs. sit: G = 0.39, *p* < 0.074; bipedal vs. stand: G = 7.93, *p* < 0.018). Cling was infrequently used (Figure 2). While clinging, Acacia rats used small and vertical substrates at very high rates (Table 4; substrate size, cling vs. stand: G = 20.6, *p* < 0.001; cling vs. sit: G = 7.9, *p* = 0.021; substrate inclination, cling vs. stand: G = 110.8, *p* < 0.001; cling vs. sit: G = 75.8, *p* < 0.001). Hang was rare and primarily involved the use of all limbs (57.1% of hanging postures). Hang largely occurred below small substrates of mainly horizontal and vertical inclinations (Table 4; substrate size, hang vs. stand: G = 14.1, *p* < 0.001; hang vs. sit: G = 7.8, *p* = 0.023; substrate inclination, hang vs. stand: G = 86.2, *p* < 0.001; hang vs. sit: G = 99.8, *p* = < 0.001; hang vs. cling: G = 57.5, *p* < 0.001).

## 4. Discussion

The present study, based on behavioral observations of captive animals, shows that Acacia rats are primarily arboreal, employing a diverse locomotor repertoire composed of considerable rates of quadrupedal, climbing, clambering activities and gap-crossing modes. Furthermore, they frequently used standing postures, but less frequent rates of sit, bipedal stand and cling. Most positional modes were associated with fine and horizontal substrates. Small substrates were strongly preferred during both locomotion and postural behavior, whereas medium and especially large substrates were avoided, particularly in postural contexts. On the other hand, horizontal substrates were consistently preferred, while oblique and vertical supports were avoided during postural activity and only used opportunistically during locomotion. The positional patterns observed in this study broadly align with the known ecological and morphological characteristics of Acacia rats. Nevertheless, as observations were conducted under captive conditions, behavioral expression was inevitably influenced by the structural properties and spatial limitations of the enclosure. Although the enclosure was designed to approximate natural habitat conditions, it could not replicate the ecological complexity of wild arboreal environments, including variation in resource distribution, predator presence, canopy discontinuities, and the diversity of mechanical constraints encountered in natural substrates. Consequently, extrapolations to wild behavioral patterns should be interpreted cautiously, as the architecture of the enclosure indubitably shaped these behavioral strategies.

The selective exploitation of fine, horizontal substrates is usually related to small-bodied arboreal specialists that forage and move along the terminal branch networks [2,3,16,17]. This pattern is consistent with observations from other small arboreal rodents, such as the African woodland dormice [37] and Eurasian harvest mice [51], which similarly exploit fine peripheral substrates that provide access to food sources while limiting access to potential predators. These types of arboreal substrates may offer an optimal compromise between stability and accessibility, especially for small-bodied mammals, as the relative instability of such substrates decreases with body size [50,52,58]. Thus, the strong preference for small horizontal substrates in Acacia rats may be related to a functional adaptation to terminal branch environments within Acacia tree canopies [18,21,41,44,45,46,47,49].

The marked avoidance of medium and large substrates, particularly during postural behavior, provides additional evidence that Acacia rats are specialized for fine-branch use. In general, wider substrates provide relatively stable transit routes within the canopy and can be used by a broad range of arboreal and scansorial mammals of varying body sizes [2,37]. Such supports typically function as primary pathways for movement and, in many species, as platforms for resting or feeding. The consistent avoidance of larger substrates by Acacia rats, even during postural modes, indicates that these animals do not rely on such stable substrates, but instead preferentially exploit finer parts of the canopy. This pattern most likely suggests a high degree of specialization toward terminal branch microhabitats, where stability is reduced, but access to food resources and reduced competition or predation risk may provide significant ecological advantages. Previous experimental studies have further underscored the capacity of Acacia rats to effectively use fine substrates by adjusting their gait patterns [50].

Acacia rats demonstrated a highly diverse positional repertoire, with considerable rates of different locomotor and postural modes. However, quadrupedalism dominated the locomotor repertoire. This mode is typical of small arboreal mammals, where symmetrical gaits provide enhanced stability on narrow substrates [34,50,58,59]. The strong association of quadrupedalism with small and horizontal substrates indicates that this locomotor mode represents an efficient and active strategy for exploiting terminal branches.

Climbing constituted the second most frequent locomotor mode and was strongly associated with small, vertical substrates. Climbing is essential for rapid vertical displacement within tree-crowns and enables the exploitation of tree canopies. The Acacia rats’ recurved claws, semi-prehensile feet equipped with raised pads and a well-developed, partially opposable fifth toe [18,49] functionally facilitate the frequent use of vertical substrates of different diameters, enabling rapid and efficient vertical ascents [17,60]. Moreover, when moving across multiple narrow substrates, usually located at the edges of tree-crowns, Acacia rats seem to shift to clambering, which is primarily associated with small and oblique substrates. The use of clambering bouts suggests a flexible locomotor strategy for negotiating complex branch networks, particularly in structurally discontinuous canopies. Similar behaviors have been reported in arboreal and scansorial rodents that are able to exploit both a wide range of arboreal and terrestrial environments [61]. These strategies are more likely related to the exploitation of the inverted conical canopy architecture of Acacia trees, which provides both refuge and access to specific food resources [21,49].

The arboreal dexterities of Acacia rats are further supported by the balanced use of bridging and leaping during gap-crossing behavior. Bridging behavior, which dominated on small horizontal supports, likely minimizes energetic cost and reduces the risk of falling by enabling the crossing of short gaps and most likely decreasing the use of long meandering routes within the canopy. On the other hand, leaping allowed access to more distant small and horizontal substrates. The structural properties of Acacia crowns, characterized by fine, sparsely connected branches, likely play a critical role in shaping similar behaviors [47]. In such environments, frequent short-distance bridging may be more advantageous than long-distance leaping, particularly for small mammals, which are not morphologically adapted to long leaps (i.e., lacking long hind limbs that promote thrust for liftoff). Occasional use of leaping, as observed here, may therefore represent a context-dependent response to structural constraints.

Postural behavior was dominated by stand, followed by sitting and bipedal postures. All these modes were strongly associated with small and horizontal substrates, indicating that these supports provide stable platforms for feeding, resting, and exploring the environment. The relatively low frequency of clinging and hanging postures suggests that suspension-based behaviors play a minor role in the postural repertoire Acacia rats. When present, these postures were strongly associated with vertical or inverted substrates, indicating their use in specific microhabitats (peripheral branches) and specific contexts (acquisition of otherwise inaccessible resources). This behavior contrasts with more suspensory arboreal mammals, such as Eurasian harvest mice [51] or bare-tailed wooly opossums [62], where hanging behaviors are more frequent and functionally essential for negotiating the canopy.

The behavioral patterns observed in this study are generally consistent with the known ecological and morphological characteristics of Acacia rats. The species’ association with large Acacia trees, which function as habitat islands in savanna ecosystems [47,48], likely imposes strong selective pressures for efficient movement within fine-branch networks. The species demonstrated increased use and preference for small and horizontal substrates in almost all contexts and modes. This approach is supported by their short robust limbs and broad semi-prehensile hind feet [18,49] and by decreasing speed and reducing the aerial phase of their stride while favoring more lateral gaits [50]. This strategy most likely facilitates access to and acquisition and manipulation of food resources such as fruits, seeds, and arthropods located on terminal branches. On the other hand, the frequent use of climbing and clambering activities is further assisted by their ability to lower their speed and adopt a statically stable mode of locomotion, enabling secure upward movement from ground burrows to the outermost branches [50]. In doing so, they exploit the inclined architecture typical of Acacia trees [17,18,21,38,41,42,45,47,49].

Morphological studies of arboreal rodents have demonstrated that traits such as elongated digits, flexible joints, and specialized limb proportions are linked to climbing and grasping abilities [63]. However, recent broad comparative analyses indicate that many rodents, especially small-bodied species, occupy a relatively generalized postcranial morphospace associated with a flexible, multi-functional appendicular design [64,65]. Thus, the extent to which these traits translate into behavioral specialization can vary. Recent work suggests that functional diversity in mammalian postcrania is influenced not only by the locomotor mode but also by body size and ecological constraints [66]. In small-bodied species like Acacia rats, this may result in a combination of moderate morphological adaptation and strong behavioral specialization. In effect, the interplay between the morphological traits, associated with arboreality, such as the long tail covered with elongated tactile hairs, the relatively short but robust limbs, the recurved claws, the broad hind feet with raised pads and short toes, and the well-developed, partially opposable fifth toe [14,15,16,18,35,49,67], and corresponding positional patterns may represent a primary mechanism for exploiting arboreal niches and most likely enables Acacia rats to negotiate effectively substrates of varying sizes and inclinations within Acacia trees.

These positional adaptations are likely crucial for the survival of Acacia rats, which are concentrated in patchily distributed tree clusters within the arid and nutrient-poor landscapes of the African savannah. These conditions attract a wide range of savannah fauna, intensifying interspecific competition [47]. The combined morphological and behavioral traits of Acacia rats enable them to effectively exploit the microhabitats associated with Acacia trees, for nesting, shelter among thorns, burrowing near roots, and feeding on plant materials and arthropods found in and around the trees [17,18,21,38,41,42,45,47,49]. The effective use of these environments highlights the ecological importance of Acacia rats, serving as prey, parasite hosts [21,68], and agents of seed dispersal [69]. As such, Acacia rats may represent a key species in maintaining Acacia-dominated ecosystems, which represent vital components of the African savannah [70]. Large Acacia trees are critical habitat elements [47,48]. Removal of mature trees may directly affect nesting availability and arboreal pathways. Because Acacia rats depend on structurally complex arboreal networks for predator avoidance [18,21], habitat degradation could disproportionately impact the species. Thus, understanding how Acacia rats adapt to and overcome the challenges of their habitat is therefore essential. This study contributes to that understanding by examining the adaptive value of their positional strategies and aims to provide a foundation for future research on this ecologically significant yet relatively understudied species and its habitat.

## 5. Conclusions

Overall, the locomotor and postural behavior of Acacia rats demonstrate clear functional adaptation to arboreal life in structurally complex but discontinuous savanna canopies. The strong preference for fine horizontal substrates, combined with efficient climbing and controlled gap-crossing strategies, supports its classification as a specialized arboreal rodent. At the same time, the broader context of rodent ecomorphology suggests that this specialization is achieved primarily through behavioral mechanisms rather than extreme morphological differentiation. This highlights the importance of integrating behavioral, morphological, and ecological data in understanding the evolution of arboreality in small mammals.

Although captive environments provide valuable opportunities for controlled behavioral observation, they inevitably constrain the range of available substrates and ecological contexts. However, by quantifying substrate availability and comparing it with observed use, the present study was able to distinguish true preference from environmental constraint. Future studies should aim to validate these findings under natural conditions, incorporating field-based observations, kinematic analyses, and ecological measurements. More particularly, investigating seasonal variation, predator avoidance behavior, and resource distribution would provide further insights into the adaptive significance of arboreal locomotion in Acacia rats.

## Figures and Tables

**Table 1:** Definition and description of the recorded variables for captive *Thallomys paedulcus*.

**Substrate type**	
Branch	Tree branches of different diameters
Artificial	Nest box, hanging feeders, window glass, metallic bars, wire mesh
Ground	Bedding of enclosure floor
**Substrate size**	
Small	Substrate fully grasped by foot
Medium	Substrate partly grasped by foot
Large	Substrate cannot be grasped by foot
**Substrate inclination**	
Horizontal	Angle between 0 and 22.5°
Oblique	Angle between 22.5 and 67.5°
Vertical	Angle between 67.5 and 90°
**Locomotor modes**	
Quadrupedalism	Symmetrical slow or moderate (walk), symmetrical fast (run) and asymmetrical fast (bound) progression along single horizontal and moderately inclined substrates
Climb	Symmetrical quadrupedal or asymmetrical bounding ascent or descent along vertical or steep substrates
Clamber	Irregular pronograde or semipronograde quadrupedal locomotion across multiple substrates of different inclinations
Leap	Gap-crossing mode involving an airborne phase with the body held horizontally or inclined, directed upwards, horizontally, or downwards, also involving hopping
Bridge	Gap-crossing mode involving reaching action across distantly located substrates with irregular limb movements
**Postural modes**	
Stand	Pronograde quadrupedal posture with either strongly flexed or semi-extended limbs
Sit	Orthograde or leaning seated posture with strongly flexed hind limbs
Bipedal	Semi-orthograde standing posture on the two semi-extended or strongly flexed hind limbs
Cling	Orthograde gripped posture with extremely flexed limbs and the head upwards or downwards on vertical or steep substrates
Cantilever	Grasping feet secure the lower part of the body to a substrate while the trunk and forelimbs are extended horizontally
Hang	Suspensory posture below a substrate using all four limbs or using the hind limbs only

**Table 2:** Percentages of use of substrate size and inclination by captive *Thallomys paedulcus*.

	Locomotion (%)	Postures (%)	Total (%)
Substrate size			
Small	66.7	68.4	67.9
Medium	13.5	24.7	21.8
Large	19.8	6.9	10.3
Substrate inclination			
Horizontal	47.5	71.8	65.5
Oblique	29.6	22.5	24.3
Vertical	22.9	5.7	10.2
*n*	1389	3887	5276

**Table 3:** Percentages of use of substrate size and inclination in the different locomotor modes by captive *Thallomys paedulcus*.

	Quadrupedalism (%)	Climb (%)	Clamber (%)	Bridge (%)	Leap (%)
Substrate size					
Small	62.0	72.9	93.8	52.6	51.5
Medium	13.7	17.0	4.9	25.1	26.3
Large	24.3	10.1	1.3	22.3	22.2
Substrate inclination					
Horizontal	63.3	0.0	33.5	69.6	76.8
Oblique	36.7	9.7	50.6	26.8	15.1
Vertical	0.0	90.3	15.9	3.6	8.1
*n*	708	306	163	112	99

**Table 4:** Percentages of use of substrate size and inclination in the major postural modes by captive *Thallomys paedulcus*.

	Stand (%)	Sit (%)	Bipedal (%)	Cling (%)	Hang (%)
Substrate size					
Small	66.2	56.2	90.2	81.2	88.1
Medium	26.6	32.7	8.6	14.7	4.8
Large	7.2	11.1	1.2	4.1	7.1
Substrate inclination					
Horizontal	74.2	86.3	84.5	0.5	38.1
Oblique	25.8	13.7	15.5	10.2	26.2
Vertical	0.0	0.0	0.0	89.3	35.7
*n*	3017	328	297	197	42

## Data Availability

The datasets presented in this article are not readily available because they are part of an ongoing study. Requests to access the datasets should be directed to D.Y.
